# Intermittent left bundle branch block

**DOI:** 10.3402/jchimp.v2i3.19037

**Published:** 2012-10-15

**Authors:** Mansoor Mozayan, Marc Mugmon

**Affiliations:** Department of Medicine, MedStar Union Memorial Hospital, Baltimore, MD, USA

**Keywords:** intermittent rate dependent bundle branch block, propofol induced bradycardia

## Abstract

Presenting electrocardiograms of intermittent left bundle branch block in a 60-year-old female.

A 60-year-old female with a history of intermittent left bundle branch block (LBBB), hypertension, and dyslipidemia underwent a routine outpatient colonoscopy. On the morning of the procedure she was afebrile and normotensive. Electrocardiogram revealed sinus bradycardia at a rate of 54 and a narrow QRS (Fig. 1A). Propofol sedation was administered. Her heart rate slowed further, down to 38. The procedure was terminated, and the propofol drip was discontinued.

**Fig. 1:**
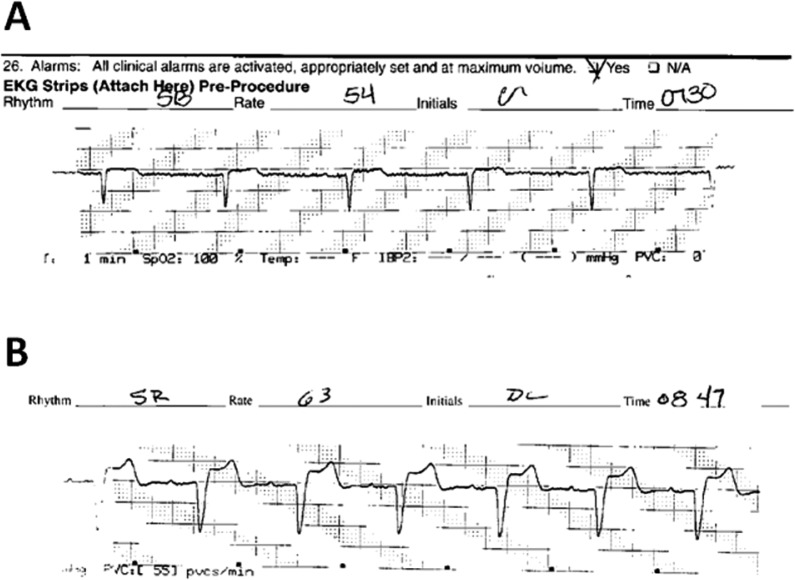
Telemetry strip obtained preprocedure (A) showing narrow complex rhythm with a heart rate of 54 and immediately after arrest (B) showing wide complex rhythm at a heart rate of 63.

One minute later the patient became asystolic and CPR was initiated. After a minute of CPR her pulse was reestablished with a rate of 63. At this time, LBBB was observed on the monitor (Fig. 1B). Myocardial infarction was considered in the differential diagnosis. Metoprolol 5 mg intravenously was administered. Electrocardiogram revealed sinus bradycardia with a narrow QRS (Fig. 2).

**Fig. 2:**
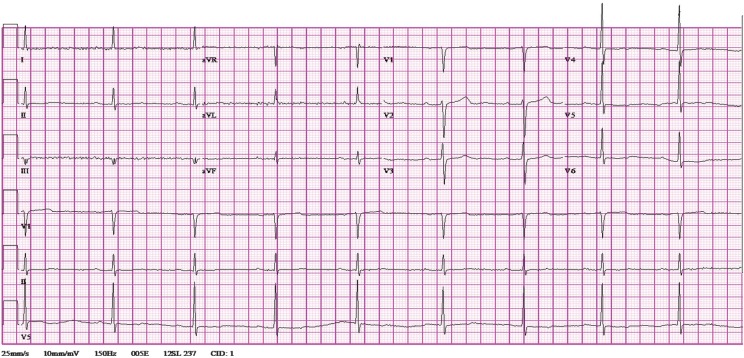
12-lead EKG obtained after metoprolol administration demonstrating normal conduction at heart rate of 53.

On examination the patient was anxious but completely asymptomatic. Other than bradycardia, with rates varying between 47 and 52, and mild hypertension (blood pressure 150/71), physical exam was unremarkable.

Three and a half hours after the administration of metoprolol, heart rate increased to 64 and EKG revealed intermittent LBBB, with both narrow and wide QRS complexes, despite a stable heart rate (Fig. 3). Fifteen minutes later, the heart rate increased to 70 and LBBB was constant.

**Fig. 3:**
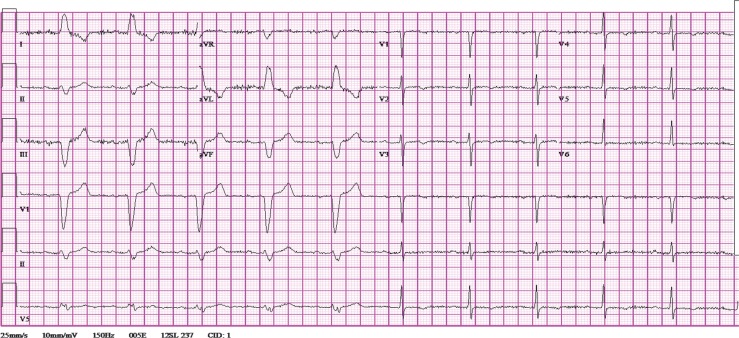
12-lead EKG obtained 3 hours after metoprolol administration at a heart rate of 63 demonstrating LBBB and normal conduction.

The patient was monitored overnight and no further events were noted. Except for a negligible rise in troponins to 0.017 (normal <0.015) and mild renal insufficiency (creatinine 1.24), her blood tests, including complete blood count and biochemical profile were normal. Echocardiogram showed a mildly dilated left ventricle with normal wall thickness and systolic function but reduced left ventricular compliance. No significant valvular abnormalities were detected.

Three weeks earlier she underwent evaluation for atypical chest pain. At that time a narrow QRS and inferior and anterior T-wave inversions were noted, while the heart rate was 49 (Fig. 4). When the rate increased to 61, LBBB recurred. Coronary angiography revealed non-obstructive coronary artery disease, indicating that the T-wave abnormalities were not due to ischemia, but, rather were due to defective intraventricular conduction (1, 2).

**Fig. 4:**
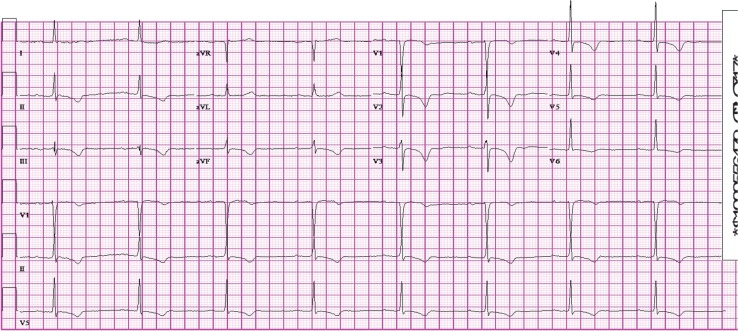
12-lead EKG obtained three weeks earlier showing first degree heart block with T-wave inversions during normal conduction at slower rate of 49.

Because of baseline bradycardia she had been initially discharged on 12.5 mg of metoprolol twice daily. However, she had been erroneously taking 25 mg of metoprolol twice daily, aggravating her bradycardia on the morning of the arrest. The arrest was attributed to propofol-induced bradycardia (3) and possibly vagal stimulation (4) during the colonoscopy.

Intermittent, rate-dependent LBBB occurs due to prolongation of the refractory period of the main left bundle branch or both the anterior and posterior fascicles together. Once the heart rate slows and the RR interval is longer than the refractory period of the conduction pathway, normal conduction can occur.

It was once felt that this prolongation of the refractory rate is usually due to intrinsic disease of the cardiac conduction pathway. However, other causes, such as myocardial dysfunction secondary to myocarditis, cardiomyopathy, left ventricular hypertrophy, valvular disease and ischemic causes need to be considered (5).

Intermittent LBBB is usually seen at higher heart rates. Interestingly, in our patient it occurred at rates as low as 61. It is important to recognize intermittent, rate-dependent LBBB and to differentiate it from ST elevation myocardial infarction or a ventricular arrhythmia.
